# Persistent use of hookah smoking among Iranian women: A qualitative study

**DOI:** 10.18332/tpc/99507

**Published:** 2018-11-20

**Authors:** Shirin Shahbazi Sighaldeh, Azam Baheiraei, Samin Dehghan, Abdurrahman Charkazi

**Affiliations:** 1 Department of Reproductive Health, Prenatal and Delivery, School of Nursing and Midwifery, Tehran University of Medical Sciences, Tehran, Iran; 2 Department of Nursing, School of Nursing and Midwifery, Tehran University of Medical Sciences, Tehran, Iran; 3 University of Medical Sciences, Tehran, Iran; 4 Health Education and Promotion, Environmental Health Research Center, Golestan University of Medical Sciences, Gorgan, Iran

**Keywords:** qualitative research, women, smoking, content analysis, hookah, waterpipe

## Abstract

**INTRODUCTION:**

Hookah smoking is the most common method of tobacco smoking among Iranian women and its rate has significantly increased over the past few decades. This study aimed to explore reasons behind persistent use of hookah smoking among Iranian women.

**METHODS:**

This qualitative study was conducted from December 2014 to March 2016. Participants were 38 Iranian women living in Tehran, the capital of Iran. They were hookah smokers at the time of this study or at least had a history of its use. Data were collected using semi-structured face-to-face interviews and were analyzed using the qualitative content analysis method.

**RESULTS:**

The main factors for persistent use of hookah smoking from the perspective of women were entertainment, a pretext to gather with old friends and family members, and a method for the creation of social networks. Hookah smoking has been described as entertainment and fun.

**CONCLUSIONS:**

Strategies aimed to curb the social issue of hookah smoking by women need to focus on the provision of appropriate entertainment methods with the consideration of advantages, such as gathering with friends and family members and consolidating relationships.

## INTRODUCTION

There is a growing tendency towards hookah smoking among college students and women. A national study, in 2007, indicated hookah as the most common form of tobacco use among Iranian women^1^. In some provinces of Iran, the prevalence of hookah smoking among women is the same as that among men^2,3^ or at least near to that of men^4^.

According to the findings of a Tehran heart and lipid study, in 2004, 63% of male students and 47% of female students had a history of hookah smoking^5^. The prevalence of cigarette smoking among male and female dentistry students who were studying in seven medical sciences universities across Iran was 9% and 6%, respectively, but the prevalence of hookah smoking was 23% and 19%, respectively^6^.

The Convention on Tobacco Control of the World Health Organization has prioritized the improvement of a woman’s understanding of tobacco health-related issues^7^. Also, reproductive health survey studies with the aim of evaluating a wide range of issues regarding reproductive health in low- to middle-income countries have called for prioritizing the hookah smoking issue in future studies^8^.

According to the WHO, an understanding of factors influencing the beginning and persistence of tobacco use and its gender-related patterns can optimize efforts to control tobacco use in any society^7^.

Medical science literature shows that contact with synthetic chemical substances may cause some changes in the woman’s reproductive behavior and even endanger fertility and lead to placenta previa, placenta abortion and premature rupture of membranes^9^. Therefore, girls and women in different ages and contexts must be involved in prevention and cessation programs of tobacco^7^.

It is known that tobacco smoking by women has nearly as many risks as men, but compared to men they are more susceptible to some lung cancers^10^. In this respect, it is predicted that in the next 10 years the prevalence of chronic obstructive pulmonary diseases (COPD) in women will surpass that in men^11^.

Given the fact that hookah smoking is the most common form of tobacco use among Iranian women, and the importance of understanding factors influencing its pattern of use among men and women, there is a need to conduct studies with a focus on behavioral patterns of tobacco smoking in women. The findings of such studies can help in the development of preventive strategies to curb the issue of tobacco smoking in women.

Currently, a few studies have been conducted on tobacco smoking among Iranian women using a quantitative design. For example, the papers of Baheiraei et al. have reported the factors associated with the initiation of hookah smoking among women, but they have provided no information about the factors associated with its persistence. Therefore, this study aimed to explore reasons behind persistent use of hookah smoking among Iranian women^12-14^.

## METHODS

### Participant recruitment

This qualitative study was conducted between December 2014 and March 2016. Participants were 38 Iranian women living in an urban area of Iran, Tehran. Using purposive sampling and snowball technique, those women who were hookah smokers at the time of this study or at least had a history of hookah smoking were chosen. They were sampled from universities, hospitals, and leisure centers, parks and traditional restaurants, following a snowball technique where one person would put the researcher in touch with her friends, colleagues, and other contacts who smoked hookah. The maximum variation in sampling was considered in terms of educational and social levels and geographical characteristics, without consideration of the participants’ age and the pattern of hookah smoking.

### Data collection procedures

The participants were asked to fill out a researcher-made questionnaire consisting of 13 questions on sociodemographic characteristics and 5 questions on their hookah smoking pattern. Also, in-depth individual semi-structured interviews were held for gathering data. The interviews were conducted by two persons who had experience and skills in conducting qualitative studies and face-to-face interviews. The interviews lasted between 30 and 90 minutes on average. The participants’ voice was recorded by a tape-recorder, except for one person who did not give permission for tape-recording. Therefore, the data of this participant were collected through notes. The convenient places for holding the interviews chosen by participants were their workplaces, homes, hospitals, and resorts. The foci of the interviews were as follows: ‘What are the reasons behind hookah smoking and its persistence by women?’, and ‘what are your perspectives and experiences of hookah smoking?’. In continuation, probing questions such as ‘will you explain more’ and ‘what do you mean?’ were asked to follow their thoughts and increase the depth of the interviews.

The interviews continued until data saturation was reached. Data saturation was reached when no new data collected added to the variations of our findings^15^.

### Data analysis

Data analysis using the content analysis method was conducted concurrently with the data collection. It meant that after holding an interview with a participant, the collected data were analyzed and then the next interview took place with another participant. Content analysis was used in this study to analyse data, develop categories and obtain an exploratory description of the study phenomenon. According to the literature, the development of categories as the expression of the manifest content is one of the core features of qualitative content analysis. For data analysis, the interviews were transcribed *verbatim* and were read several times to obtain the sense of whole. The *unit of analysis* and *meaning units* were considered the whole interview’s text and those parts regarding the experiences of participants of hookah smoking, respectively. Next, the meaning units were condensed, abstracted and labelled with codes. The codes were compared in terms of similarities and differences.

### Ethical considerations

This study research proposal was approved and funded by the research council affiliated with Tehran University of Medical Sciences, Tehran, Iran that corroborated its ethical considerations and provided a grant (no. 91-03-61-20040).

The participants were provided with information about the study method and their anonymity throughout the research process. They were informed that participation in this study was voluntary and they could withdraw at any time without being penalized. Also, permission to tape-record the interviews was obtained. The people who agreed to participate in the study were requested to sign a written informed consent form.

## RESULTS

### Sample characteristics

The range of the participants’ age was from 21 to 39 years with a mean of 30 (SD=5.38) years. The mean of the age for hookah smoking onset was 16.5 (SD=3.23) years with a range of 7–26 years. The participants were single and married, from different ethnic groups, and lived in different geographical locations in Tehran, Iran. Half of the women were employed. They had a range of educational degrees from Diploma to a Master’s degree. The main hookah smoking pattern of the women was described as ‘occasionally’. More details of the participants’ demographic characteristics are presented in Table 1. The data analysis led to the development four main categories: 1) leisure, entertainment and fun; 2) hookah smoking by family members and relatives; 3) convening under the pretext of hookah smoking, but for the sake of benefiting all; and 4) the impact of friendly gathering on persistent use of hookah. These are also shown in Table 2.

**Table 1:** The demographic characteristic of the participants

	*N*	*%*
**Age (years)**		
10–15	2	13.4
16–20	3	20
21–25	6	40
26–30	4	26.6
**Marital status**		
Single	10	66.6
Married	5	33.4
Divorced	-	
Widow	-	
**Occupation**		
Employed	6	40
Unemployed	9	60
**Ethnicity**		
Farse	11	73.4
Kord	1	6. 6
Lor	-	
Tork	3	20
**Educational level**		
Academic	9	60
Diploma	6	40
High school	-	
Primary school	-	
**Residence in Tehran**		
North	1	6.6
South	4	26.6
West	5	33.4
East	2	13.4
Center	3	20
**Pattern of smoking**		
Only occasional	9	60
Occasional or daily	1	6.6
Occasional or weekly	2	13. 6
Daily or weekly	1	6.6
Every two weeks	1	6.6
Twice a week	1	6.6

**Table 2:** Categories derived from qualitative data

1. Leisure, entertainment and fun
2. Hookah smoking by family members and relatives
3. Convening under the pretext of hookah smoking, but for the sake of benefiting all
4. The impact of friendly gathering on persistent use of hookah

### Leisure, entertainment and fun

Most participants described hookah smoking as entertainment and fun. They often engaged in hookah smoking to have some fun. They spent their time with friends, relatives and family members during hookah smoking. Having no other type of fun was the main reason behind smoking hookah:

*‘It [hookah smoking] is 99% for having fun and 1% for other reasons. Since I have no other type of fun in the society, I am pulled after it*… .’ (P 1).

Some participants believed that variations in the location of hookah sale, type of substances and ingredients incorporated into the hookah, and its price, led to easy and affordable access to hookah by different people. In addition, some people would be able to smoke hookah at home, if they could afford buying a pack of tobacco and a hookah set. Moreover, other types of entertainment with higher prices could not be often affordable by all:

*‘There are different places around here for buying hookah. In some places, it is expensive and in some other places it is so cheap. It means that I can smoke hookah by whatever prices affordable for me.’* (P 9).

Restaurants as the main places where hookah would be sold were very attractive and pleasant places for hookah smokers. While other types of entertainment had determined timing schedules and users needed to leave the venues after closing time, restaurants had no determined closing time and were always open. In restaurants, people could sit on the ground or chairs and smoke hookah according to the Iranian tradition:

*‘Restaurants are always open. I prefer to go a restaurant and spend my times there. When my friends and I sit on the stool rather than ordinary chairs, the feeling of intimacy is increased between us.’* (P 6).

### Hookah smoking by family members and relatives

One of the main reasons behind the persistent use of hookah smoking was reported to be the tradition of hookah smoking by Iranians at familial parties. Some participants noted that hookah was traditionally smoked by their family members. Fathers, mothers, sisters, brothers, second- and third-degree relatives were mostly hookah smokers in the family. However, it had become more popular among young men and women as a new type of entertainment:

*‘My sisters accompany me, when I decide to smoke hookah.’ (P 9), ‘My cousins and I convened together and smoked hookah.’ (P 3), ‘My brothers are cigarette smokers. If I smoke hookah, they come along and smoke it as well as my sister… we are rivaling for smoking hookah.’* (P 13).

Common places for hookah smoking were familial parties at home, parks and other resorts. Family gatherings and access to hookah at these gatherings were mentioned as the most important reason for hookah smoking:

*‘For the first time, I smoked hookah in a familial party…. men and women would smoke hookah. Except my father that smoked cigarette time to time, my family members do not smoke cigarette or smoke hookah. My father’s relatives mostly smoke hookah, but not my mother’s relatives. I smoked hookah just at home or outgoings.’* (P 2).

The effect of hookah smoking by the adult members of the family, such as the father and mother, on the younger members’ inclination to smoke hookah was emphasized. One participant stated that hookah smoking by her grandmother made her attitude positive toward hookah smoking:

*‘My grandmother was used to hookah smoking since she was in the age. Once, she said: “I had no negative attitude toward hookah smoking, because my mother smoked it also. I had no a bad feeling about it”.’* (P 11).

### Convening under the pretext of hookah smoking, but for the sake of benefiting all

According to the participants, ‘being together’ was given as the main motivator for hookah smoking. Hookah smoking with others was described as a very pleasant experience, whereas being alone was not appealing at all for smoking hookah. To smoke hookah, someone would take the lead and invite others to come along to hookah smoking. They believed that hookah smoking was a satisfying partnership with others:

*‘Hookah smoking provides the opportunity to be together with friends and relatives. We enjoy our time laughing together…I smoke hookah in gatherings… for instance, this is correct that hookah is shared between two persons, but the satisfaction belongs to both of them.’* (P 9).

It was believed that hookah smoking was a pretext to gather people together. The underlying reason was a need for being loved and supported by others. In fact, hookah smoking was a pretext for meeting friends:

*‘We go for laughing…friends and families want to see each other and ask about each other…therefore, hookah smoking is only a pretext. The real reason is being together. When I meet my friends, my childhood memories are refreshed. Sometime hookah is available, but I do not smoke it, because I am immersed in discussions with others about different matters. Such gatherings are so relaxing, because we support each other and share our friendship.’* (P 8).

### The impact of friendly gathering on persistent use of hookah

The participants stated that gathering friends together created favorable conditions for hookah smoking:

*‘This is about three months that I have not smoked hookah at all and I do not care about it. However, if I get in the situation for instance in a gathering with friends, I smoke hookah.’* (P 9).

If someone was put in a friendly gathering, even if she was not interested in smoking at all, she might smoke hookah, because friends rejected those who did not behave according to the group norms:

*‘Sometimes I am not in mood to smoke hookah, but I do it for not being rejected by friends.’* (P 9).

It was stated that the important characteristic of the group that gathered to smoke hookah was that all members were interested in hookah smoking. It meant that hookah was not served at gatherings where some persons were not hookah smokers:

*‘If I am with a friend who is not interested in hookah smoking, I do not smoke hookah. To enjoy being together, when I want to smoke hookah, I only invite those who like smoking hookah to not remain alone.’* (P 1).

## DISCUSSION

This study explored the factors influencing the persistent use of hookah smoking among Iranian women. In this respect, having fun was mentioned as the most important factor for persistent use of hookah. Accordingly, a lack of appropriate social entrainment for women, easy access to hookah smoking in restaurants, and cheap price were mentioned as the reasons for frequent hookah smoking by the women. Other Iranian studies confirm that people’s interest in hookah smoking stemmed from being deprived from healthy entertainment and a need to be amused and have fun^4,16-18^.

Some other studies introduced the socio-psychological motivators influencing hookah smoking^19,20^. The Amin et al. study with school age boys and girls showed that the most common places where hookah was smoked were gatherings with friends and family members, for entertainment^21^. According to a qualitative study by Baheiraei et al., Iranian women’s needs for entertainment and amusement were important reasons for beginning hookah smoking^13^.

Being together under the pretext of hookah smoking, but for benefiting all was another reason for the persistent use of hookah. Also, hookah smoking was described as social entertainment for Iranian families that increased their feelings of intimacy and friendship. When family members and relatives gathered, once upon a time, they enjoyed being together rather than smoking hookah. Therefore, hookah smoking as a pretext to gather together could consolidate familial friendship and provide psychological support for each other. Maziak et al. in their study with students reported that hookah smoking was a strategy to create social networks and that rarely hookah was smoked outside social environments. Also, the students started hookah smoking to communicate with others^22^. According to the study by Baheiraei et al., hookah smoking in a social environment had an important role in women’s temptation to start smoking hookah. The authors emphasized that being together is often for other purposes rather than hookah smoking, but gatherings could indirectly influence the onset of hookah smoking^13^. According to our study, gatherings had a direct relationship with hookah smoking persistence, because enjoying being together was intertwined with smoking hookah. Therefore, benefiting from gatherings at least needs hookah smoking once and has a relationship with its persistence.

Hookah smoking by the adult members of the family, parents, husband, sisters, brothers, and peers, could also lead to hookah smoking persistence by the women in our study. Hookah smoking often took place in familial gatherings, when family members and relatives gathered at home or visited resorts, and not when the participants were alone. Iranian women often face many restrictions with regard to cigarette smoking, but families easily approve hookah smoking as traditional entertainment, and girls are allowed to use it inside and outside the home^23^.

Some participants believed that easy access to hookah at home could lead to more hookah smoking. Other studies reported that hookah smoking at home was a positive predictive factor for the persistence in its use^23-26^.

In our study hookah smoking in the family was an associate factor influencing persistence in its use and a factor influencing its onset^12,14^. Therefore, hookah smoking in the family environment can both initiate its use and encourage its persistence.

We found that hookah smoking by adult family members encouraged use by younger members. However, it appears that it mainly influences the onset of hookah smoking. Baheiraei et al. stated that positive attitudes by the family, society and people toward hookah smoking encouraged others to start using it^12^. According to another study there are relationships between positive attitudes toward hookah and the prevalence of its use^27^.

Our study participants also emphasized the influence of friends and friendly gatherings on hookah smoking persistence. For instance, the women stated that they used hookah mainly in friendly gatherings. It meant that friends motivated each other to smoke hookah. According to international literature, hookah smoking was considered a social event, entertainment and fun by friends^18,28^. Therefore, friends’ influence for hookah smoking was more important than that for cigarette smoking^29^. According to Maziak et al., college students started smoking hookah when they became interested in the creation of social networks. Therefore, hookah smoking became one unavoidable part of students’ entertainment^22^.

Baheiraei et al. described the role of boy and girl friends and their influence on the onset of hookah smoking. They often gathered in restaurants and encouraged each other to smoke hookah. In this respect, the development of friendship with hookah smokers created a potential opportunity to start smoking hookah^14^. Other studies also confirmed the roles of friends in the onset of hookah smoking as well as its impact on the persistent use of hookah^4,23,30-32^.

A limitation of this study was that some women and girls would be gathered at homes, restaurants and resorts to use hookah and so were not interested to be interviewed. Therefore, the researchers used the snowball-sampling method to attract the women’s trust and recruit as many as possible participants. Also, hookah smoking by women was against the Iranian social norm. Therefore, the participants might have censured their words. To resolve this issue and collect in-depth data, the interviewer arranged personal and private interviews, and assured them of the anonymity of their identities and confidentiality of the findings.

## CONCLUSIONS

We found that hookah smoking was considered as an entertainment method for the Iranian women. It was also used for amusement with their friends, relatives and family members. Moreover, hookah smoking was a pretext to gather with old friends and family members, as well as a method for the creation of intimate social networks. These are the main factors for the persistent use of hookah among Iranian women. Therefore, strategies aimed to curb the social issue of hookah smoking by women need to focus on the provision of appropriate and healthy entertainment methods with the consideration of advantages such as gathering of friends and family members and consolidating their relationships. In addition, due to the increasing range of waterpipe smoking among Iranian women, this public health problem needs to be also investigated qualitatively in other parts of the country.
